# Reducing inherent biases introduced during DNA viral metagenome analyses of municipal wastewater

**DOI:** 10.1371/journal.pone.0195350

**Published:** 2018-04-03

**Authors:** Nichole E. Brinkman, Eric N. Villegas, Jay L. Garland, Scott P. Keely

**Affiliations:** National Exposure Research Laboratory, United States Environmental Protection Agency, Cincinnati, Ohio, United States of America; Sidra Medical and Research Center, QATAR

## Abstract

Metagenomics is a powerful tool for characterizing viral composition within environmental samples, but sample and molecular processing steps can bias the estimation of viral community structure. The objective of this study is to understand the inherent variability introduced when conducting viral metagenomic analyses of wastewater and provide a bioinformatic strategy to accurately analyze sequences for viral community analyses. A standard approach using a combination of ultrafiltration, membrane filtration, and DNase treatment, and multiple displacement amplification (MDA) produced DNA preparations without any bacterial derived genes. Results showed recoveries in wastewater matrix ranged between 60–100%. A bias towards small single stranded DNA (ssDNA; polyomavirus) virus types vs larger double stranded DNA (dsDNA; adenovirus) viruses was also observed with a total estimated recovery of small circular viruses to be as much as 173-fold higher. Notably, ssDNA abundance decreased with sample dilution while large dsDNA genomes (e.g., *Caudovirales*) initially increased in abundance with dilution before gradually decreasing with further dilution in wastewater samples. The present study revealed the inherent biases associated with different components of viral metagenomic methods applied to wastewater. Overall, these results provide a well-characterized approach for effectively conducting viral metagenomics analysis of wastewater and reveal that dilution can effectively mitigate MDA bias.

## Introduction

Viral metagenomics have become invaluable in understanding viral abundance, discovery, quantitation, and diversity in the human gut [1, 2], marine environment [3], sewage sludge [4, 5], wastewater [6, 7] and reclaimed water [8]. The advent of next generation sequencing technologies (NGS) has now made it even easier to conduct such studies with more depth and improved accuracy on assessing viral abundance and community structures in various environmental matrices. Viral metagenomics may facilitate improved risk characterization and mitigation strategies for water reuse by defining new indicators and surrogates that are more abundant in these matrices that can be used to better assess viral pathogen occurrence and removal efficacy.

While high-throughput sequence analysis is a powerful tool for uncovering the assemblage of viruses in the environment, sample and molecular processing steps can alter the true distribution of viral groups in sample. Initial concentration steps can introduce potential sample losses, especially for enteric viruses that are prone to aggregation, particularly at low pH [9]. A better understanding of the fate of viruses and their nucleic acid contents (e.g., RNA and DNA) through this initial sample concentration/filtration step is needed in order to optimize the procedure to minimize these effects. This would result in a sample that more closely represents the true nature of the viral community structure and their relative abundances in the environment. Additionally, as with many nucleic-acid based detection analyses that are used for molecular epidemiology of viruses in the environment, removing contaminating nucleic acids, especially extraviral DNA/RNA (e.g., bacteria) is also critical [10].

One of the difficulties with conducting viral metagenomics in environmental samples is the limited amount of viral nucleic acids obtained from samples. The development of whole genome amplification, like multiple displacement amplification (MDA) [11], however, have made these types of studies possible. For example, the use of the MDA technique allowed for the investigation of the viral metagenome of marine environments, revealing a highly diverse viral community structure with *Microviridae* family being one of the dominant clades reported [3]. Although MDA techniques have been useful for analyzing samples containing limited amounts of DNA, they have been known to introduce biases [12] by preferentially amplifying single stranded viral DNA (less than 10Kb), particularly small circular DNA, like those that belong to the *Microviridae* and *Polyomaviridae* families [13–15]. The preference of amplifying small circular DNA is one possible explanation for the observed dominance of the *Microviridae* family, a small circular DNA, in certain marine aquatic environments [16]. Therefore, viral metagenomics analyses can be improved through better evaluation of MDA bias and potential mitigation approaches.

The objective of this study is to understand the inherent variability and biases introduced when conducting DNA viral metagenomic analyses of wastewater, and define a complete procedure to efficiently concentrate and recover viruses, remove extraneous extravirion contaminating DNA, mitigate biases introduced by MDA reactions, and provide a bioinformatic strategy to analyze NGS sequences for viral community analyses. Although the viral community is comprised of both RNA and DNA viruses, the latter will be the focus of this study.

## Materials and methods

**Fig 1:**
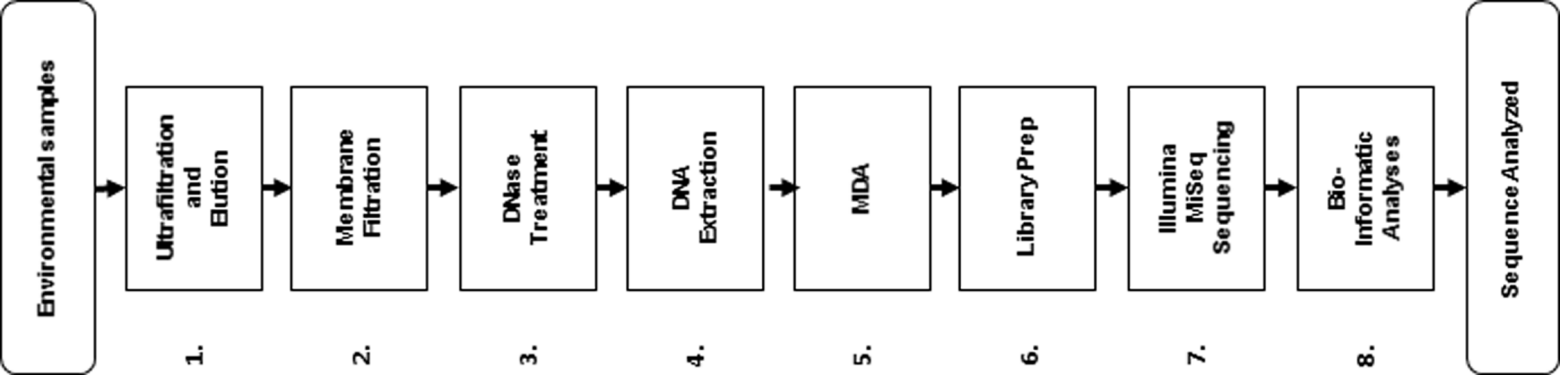
Schematic diagram of the specific procedures involved in processing wastewater samples for viral metagenomic analysis. MDA, multiple displacement amplification.

This section explains the techniques used to prepare environmental water samples for viral metagenomics analysis (Fig 1) and the experiments conducted to understand how the selected techniques may influence the distribution of virus groups observed after analysis.

### Virus representatives to evaluate processing loss/bias

Adenovirus (AdV) was used to represent the dsDNA linear viruses in experiments to evaluate the recovery efficiency of ultrafiltration and elution (Fig 1, step 1) and membrane filtration (Fig 1, step 2), the loss due to capsid destruction during DNase treatment (Fig 1, step 3), the recovery efficiency of dsDNA genomes through nucleic acid extraction (Fig 1, step 4) and bias in MDA (Fig 1, step 5). To evaluate recovery efficiency through ultrafiltration/elution and membrane filtration, endogenous AdV in raw wastewater influent was used. To investigate the effects of DNase treatment, nucleic acid extraction and MDA, a highly purified, quantified commercially available preparation of Adenovirus 5 (O.D. 260, Inc., Boise, ID) was used.

BK Polyomavirus (PyV) was used to represent the circular viruses in experiments to evaluate MDA bias (Fig 1, step 5). PyV was obtained from a commercial vendor (VR-837, American Type Culture Collection, Manassas, VA) after propagation in human cells without purification.

Male-specific and somatic coliphage were used to represent culturable members of the wastewater viral community to evaluate recovery through ultrafiltration/elution and membrane filtration. Raw wastewater influent was used as a source of the male-specific and somatic coliphage.

### Conventional wastewater treatment plant samples

Samples were collected from a conventional WWTP in the greater Cincinnati area. No specific permission was required to collect samples at the public utility and field studies did not involve endangered or protected species. This plant treats approximately 7 million gallons per day, of which 94% is from residences and 6% is from commercial entities. To evaluate DNase efficacy in a variety of wastewater products, grab samples (1L) were taken from the raw influent (n = 3) and the activated sludge tanks (n = 3) and ten liters of effluent (n = 3) was collected after UV disinfection. For sequencing analysis, 1L of raw influent wastewater was collected (n = 1). All samples were transferred to the lab on cold blocks for processing.

### Small scale membrane bioreactor system samples

A MBR was constructed for treating mined blackwater as described [17]. Briefly, the 18 l reactor was initiated with activated sludge from a local wastewater treatment plant. Two Zenon ZW-1 hollow fiber membrane modules with nominal pore sizes of 0.04 μm provided a physical barrier to microbes. Flow was drawn through the membrane at a net rate of 50 ml min^-1^. The MBR was monitored continuously to ensure proper performance, as described. Samples were collected as described with the WWTP to evaluate DNase efficacy in wastewater products: 1L grab samples were collected from the blackwater influent tank (n = 3), the mixed liquor suspended solids (MLSS, n = 3) and ten liters of MBR effluent (n = 3) was also collected. All samples were transferred to the lab on cold blocks for processing.

### Sample processing of small volume samples

Initial sample processing had 2 goals: to reduce viral particle adherence to organic material and to separate the viral community from prokaryotic and eukaryotic microorganisms. Towards this end, one liter of raw influent and activated sludge/MLSS samples were supplemented with sodium polyphosphate (Sigma-Aldrich, St. Louis, MO) to 0.01% (m/v), Tween-80 (Sigma-Aldrich) to 0.01% (v/v) and Y-30 Antifoam (Sigma-Aldrich) to 0.001% (v/v). The mixture was stirred for 30 minutes followed by stepwise vacuum filtration through membrane filters of 0.8, 0.65, 0.45 and 0.22 μm pore sizes (EMD Millipore, Billerica, MA).

### Processing of large volume samples

Ten liters of treated WWTP and MBR effluent was filtered with a single pass through a Rexeed 25S hollow fiber ultrafilter (Dial Medical Supply, Chester Springs, PA) using a Masterflex L/S peristaltic pump (Cole Parmer, Vernon Hills, IL) set at 300 RPM (approximately 840 ml min^-1^). Microbes trapped in the hollow fibers of the ultrafilter were eluted by recirculating 400 ml of a solution containing 0.01% (m/v) sodium polyphosphate, 0.01% (v/v) Tween-80 and 0.001% (v/v) Y-30 Antifoam in a clockwise direction for 1 min followed by recirculation in a counterclockwise direction for 1 min then repeated once more in both directions. The resulting eluate was filtered through a series of membrane filters as described above to remove prokaryotic and eukaryotic microorganisms.

To evaluate the impact of ultrafiltration and elution on the viral community, the recovery efficiency of AdV, somatic and male-specific coliphage were assessed. Toward this end, MBR effluent (9 l) was spiked with raw wastewater influent (1L) and samples were collected before passing through the ultrafilter and after elution of the ultrafilter, but before membrane filtration. Coliphage were enumerated using the double agar layer plaque assay described below and AdV genomic DNA was measured by MPN PCR following extraction using the QIAamp DNA Blood Maxi Kit see below).

To assess recovery efficiency through membrane filtration and potential loss of members of the viral community, raw wastewater influent was used. Samples were obtained before and after membrane filtration. Coliphage were enumerated using the double agar layer plaque assay described below.

### Somatic and male-specific coliphage plaque assay

To enumerate somatic and male-specific coliphage in samples, the double agar layer method [18] was employed. Briefly, 1 ml of sample was added to 0.7% TSA with 0.1 mg ml^-1^ nalidixic acid (Sigma-Aldrich) or 0.015 mg ml^-1^ each of streptomycin (Sigma-Aldrich) and ampicillin (Sigma-Aldrich) and 0.2 ml of a mid-log culture of the appropriate *E*. *coli* host (CN13, ATCC 700609 or HS(pFamp)R, ATCC 700891). The mixtures were then poured onto solidified agar plates containing 1.5% TSA supplemented with 0.1 mg ml^-1^ nalidixic acid or 0.015 mg ml^-1^ each of streptomycin and ampicillin and incubated at 37°C for approximately 18 hours. Plaques were then counted and recorded. When ultrafiltration was used to concentrate treated effluent samples, quantities were then back calculated to the appropriate volume and normalized per ml of original sample.

### DNase treatment to remove free DNA

Free DNA (DNA not contained in virus capsids) was removed using Turbo DNA-*free* Kit (Life Technologies) according to the manufacturer’s instructions in 100 μl reactions using the rigorous protocol.

### DNase effect on intact viral capsids

The Turbo DNA-*free* DNase treatment was evaluated to assess the potential for negative impacts on virus capsids. Adenovirus 5 stock was diluted to 10^4^ and 10^2^ virus particles μl^-1^ then treated with Turbo DNA-*free* DNase in triplicate. Triplicate untreated samples were also prepared and all samples were extracted using the QIAamp DNA Blood Mini Kit (described below) and resulting genomic copies were measured by droplet digital PCR (ddPCR, described below).

### DNase efficacy in complex wastewater samples

Removal of free DNA in samples for viral metagenomics analysis is critical so that the sequencing space is used for intact virus genomes. Wastewater samples are complex matrices and therefore, verification of DNase efficacy was warranted. This was accomplished in the WWTP and MBR wastewater samples by spiking parallel samples with a DNA target (IDT MiniGene) and evaluating its degradation by MPN PCR. A MiniGene (Integrated DNA Technologies, Coralville, IA) was constructed and consisted of the pIDTSMART-Amp vector and the sequence utilized as an internal control (HepG) for qPCR [19]. Reduction of the HepG MiniGene in wastewater was determined through MPN PCR analysis (see below).

### Nucleic acid extraction

Nucleic acids were extracted from DNase treated samples using the QIAamp DNA Blood Mini Kit (Qiagen, Valencia, CA) as described by Cashdollar et al. [20] without the use of carrier RNA. To obtain genomic DNA, silica columns were eluted 3 times with 50 μl of Buffer EB. DNA concentrations were determined using Qubit ds DNA HS Assay Kit and Qubit 2.0 Fluorometer (Life Technologies). All extracts were stored at -70°C.

For extraction of 10 ml samples, the QIAamp DNA Blood Maxi Kit (Qiagen) was used according to the manufacturer’s instructions, but substituting Buffer AVL (Qiagen) for Buffer AL. Extracted DNA was eluted from the maxi columns using 1 ml of Buffer EB, then reloading the entire eluate for a second elution.

### Digital droplet PCR

ddPCR was used to quantify the AdV and PyV products resulting from whole genome amplification reactions. This was achieved using the QX200 system (BioRad Laboratories, Hercules, CA). Primers and probe assays described for qPCR of AdV [21] and PyV [22] were optimized for use in ddPCR. The 25 μl ddPCR reactions contained Supermix for Probes (BioRad Laboratories), forward primer (AdV: 700 nmol l^-1^, GGA CGC CTC GGA GTA CCT GAG; PyV: 600 nmol l^-1^, AGT CTT TAG GGT CTT CTA CCT TT), reverse primer (AdV: 700 nmol l^-1^, ACG GTG GGG TTT CTG AAC TTG TT; PyV: 600 nmol l^-1^, GGT GCC AAC CTA TGG AAC AG), 250 nmol l^-1^probe (AdV: 6FAM-CTGGTGCAGTTCGCCCGTGCCA-BHQ; PyV: 6FAM-TCATCACTGGCAAACAT-MGB) and 5 μl of sample. Droplets were generated using the QX200 Droplet Generator (BioRad Laboratories) and PCR reactions were carried out by heating at 95°C for 5 minutes, followed by 40 cycles of 95°C for 30 seconds, 55°C for 1 and a final heat step for 10 minutes at 98°C minute in a C1000 Touch Thermal Cycler (BioRad Laboratories). Droplets were scored as positive or negative for amplification using the QX200 Droplet Reader (BioRad Laboratories) and quantities were determined using QuantaSoft Software (version 1.4, BioRad Laboratories).

### Most probable number (MPN) PCR

Degradation of the HepG MiniGene by DNase and quantities of AdV genomes in samples were assessed using qPCR. Reactions consisted of 25 μl of 1X PCR Buffer II (Life Technologies), 5 mmol l^-1^ MgCl2 (Life Technologies), 1 μl ROX dye (Invitrogen), 0.4 mmol l^-1^ dNTPs, 500 nmol l^-1^ primers (forward: GCA AGC CCC AGA AAC CG; reverse: CAA GAT GAC CGG GAT TTA CGA), 100 nmol l^-^^1^ probe (VIC-TCACCCATCCACCACCT-MGB) and 5 μl of sample. Reactions were carried out in a StepOne Plus (Life Technologies) by incubating at 95°C for 10 minutes, followed by 40 cycles of 95°C for 15 seconds and 60°C for 1 minute. Log_10_ reductions of the HepG MiniGene were assessed by determining the C_T_ difference between DNase treated and untreated samples and dividing by 3.32.

AdV MPN PCR was performed in the same manner described above, using the primers (400 nmol l^-1^) and probe (150 nmol l^-1^) described for ddPCR. Adenovirus quantities were determined using most probable number (MPN) analysis whereby samples are run at 3 dilutions (undiluted, 1:5 and 1:25) with 5 replicate reactions at each dilution. The resulting C_T_ values were used to score the reactions as positive or negative and the number of positive. The concentration of molecules per unit volume was estimated by finding the root of the partial derivative log-likelihood function:

$$\left[\frac{x_{i}z_{i}}{1-e^{-uz_{i}}}-n_{i}z_{i}\right]\sim 0$$ (1)

where *u* is the estimated number of molecules, *e* is Euler’s number, *x*_*i*_ is the number of positive PCRs of the *i*th dilution, *n*_*i*_ is the number of bernoulli trials of the *i*th dilution and *z*_*i*_ is the relative volume of the *i*th dilution [23]. R-Statistics [24] was used to iterate an approximate solution and the script is available upon request. Negative PCR controls (no template controls) consisted of using 10 mmol l^-1^ Tris-HCl, pH 8.5 (also used as the diluent for samples) and positive controls were extracts of the appropriate virus stock.

### Whole genome amplification

Whole virus DNA genomes were amplified by multiple displacement amplification (MDA) using illustra GenomiPhi V2 DNA Amplification Kit (GE Healthcare Life Sciences, Pittsburgh, PA) following the manufacturer’s instructions, but allowing the reactions to incubate overnight. MDA products were purified using QIAEX II Gel Extraction Kit (Qiagen) following the manufacturer’s protocol for desalting and concentrating DNA solutions. DNA concentrations were determined using Qubit ds DNA HS Assay Kit and Qubit 2.0 Fluorometer (Life Technologies) and stored at -20°C. AdV and PyV were used as controls in the MDA reactions. AdV5 and PyV MDA products were quantified by ddPCR (see above).

### MDA bias calculations

MDA bias was assessed by mixing a constant quantity of 1 x10^5^ genomes of PyV with increasing levels (1 x10^5^, 2 x10^5^, 4 x10^5^, 8 x10^5^ and 1.6 x10^6^ genomes) of AdV in Tris buffer before and after MDA. The performance of MDA of AdV was assessed in wastewater by spiking equal quantities (10, 100, and 1000) of AdV and PyV in primary influent and measuring AdV MDA products by ddPCR. Exponential decay was used to describe the ratio of PyV to AdV MDA products as a function of the input ratio of AdV to PyV, written as

$$f\left(x\right)=ae^{-bx}+c$$ (2)

where *f*(*x*) is the ratio of PyV to AdV MDA products, *x* is ratio of AdV to PyV input DNA, and *e* is Euler’s constant. The PyV/AdV MDA DNA ratios were normalized by

$$\hat{y}=\frac{\frac{PyV_{\left(ddPCR\right)}}{AdV_{\left(ddPCR\right)}}}{\frac{AdV_{\left(Kb\right)}}{PyV_{\left(Kb\right)}}}$$ (3)

The quantity of AdV needed to offset MDA bias was estimated by nonlinear regression of Eq 2. The offset (i.e., log_10_ (*x*)) was estimated using Past3 software [25] to determine the parameters in Eq 2 and by setting *f*(*x*) = 1 the equation was re-arranged and solved as follows

$$\log_{10}x=-\frac{\text{ln}\left(\frac{\left(f\left(x\right)=1\right)-c}{a}\right)}{b}$$ (4)

The estimate of *x* was determined by exponentiating the solution for Eq 4. Total recovery of virus was estimated using the following:

$$TotalRecovery=R_{\left(Concentration\right)}*R_{\left(DNase\right)}*R_{\left(QIAamp\right)}*R_{\left(MDAbias\right)}$$ (5)

### Influent wastewater sample processing for sequencing

For sequencing analysis, 1L of raw influent wastewater was collected (n = 1). The 1L sample was amended with detergents and filtered through membrane filters as stated above. The sample was then diluted in triplicate 10-fold series to 10^−5^ (10-fold intervals referred to as d0, d1, d2, d3, d4, and d5 for the lowest to highest dilutions). Each dilution was then subject to DNase treatment, nucleic acid extraction and MDA as described above. The range of DNA concentrations used for MDA was 30 pg, 3 pg, 300 fg, 30fg, 3fg and 0.3 fg. The MDA product libraries from each triplicate dilution was then prepared for sequencing, sequenced and analyzed as described below.

### Library preparation and sequencing

MDA products were fragmented, tagged and normalized using NexteraXT DNA Library Preparation Kit (illumina, San Diego, CA) and Nextera XT Index Kit (Illumina) as described in the manufacturer’s protocol. Paired-end sequencing (2 x 300 bp read-pairs) was performed using MiSeq Reagent Kit v3 (Illumina).

### Bioinformatic analysis

The sequence reads are available at NCBI under BioProject identifier PRJNA434744. Velvet de Bruijn graph assembler was used because it has a low chimera rate of < 5% for virus metagenomes [26]. The pair-end reads were assembled using Velvet software version 1.2.08 [27]. The virtigs (virus contigs) are publically available (i.e., project number 12194) at the MG-RAST (the Metagenomics RAST) server version 3.5 [28]. Aw et al. [6] (referred to as ‘Aw’) virtigs were retrieved from MG-RAST for re-analysis. Wastewater virtigs from this study and Aw were compared using MetaVir 2 tools (available at metavir-meb.univ.bpclermont.fr) [29] and also MG-RAST analysis. Analysis of the virtigs can be re-created at the MetaVir 2 server (i.e., “wastewater phage”). Open reading frames (ORFs) were predicted for each virtig using MetaGeneAnnotator. Circular genomes were detected using a Perl script that compares identical k-mer frequencies at the two ends of each virtig. Translated virtig ORFs were then compared to several databases, including the RefseqVirus protein database from the NCBI (using BLASTP and e-value threshold of 10^−3^) and the PFAM database of protein domains (version 26.0) using HMMScan (with a threshold of 30 for the score). A direct comparison of ORFs within a viral community was also computed through a BLASTP (e-value threshold of 10^−3^). Using the BLASTP results against reference viruses, three types of taxonomic compositions were computed: (i) best hit affiliation of each predicted virtig gene, (ii) best hit affiliation of each virtig, and (iii) lowest common ancestor (LCA) affiliation of each virtig. The LCA affiliation was designed to integrate multiple hits on a single virtig. Thus, 1 to 5 affiliated genes were evaluated and the affiliation was made at the highest taxonomic level.

Non-metric multidimensional analysis of nucleotide composition bias was performed using MetaVir 2, the viral community in this study and the viral community of Aw et al. [6].

Hierarchical classification using SEED Subsystems (function level) available at MG-RAST [28] was used to describe the gene proteins in the undiluted d0 virtigs. The criteria used for this was a maximum e-value of 1e-5, a minimum identity of 60%, and a minimum alignment length of 15 measured in amino acids for protein. DESeq was used for normalization. Rarefaction analysis was conducted after trimming the fastq files with Trimmomatic software [30] and then joined with Fastq-join [31]. Tblastx was performed on the filtered read-contigs and the blast table was loaded into MEGAN6 Community Edition (version 6.5.7, built 7 Sept 2016) software. MEGAN6 was used to perform rarefaction analysis of 10,000 read-pairs randomly selected from each dilution.

### Statistical analyses

PCA and Biplot analysis of a correlation matrix of the scores and loadings was performed using Past3 software [25]. Maximum length of the virtigs, number of virtigs, N50, GC content of the virtigs were determined with MetaVir2 tools. Mann-Whitney Rank Sum Tests and ANOVA were performed in SigmaPlot (version 13, Systat Software, Inc., San Jose, CA).

## Results

### Recovery efficiency through ultrafiltration and membrane filtration

Ultrafiltration and elution were evaluated to assess recovery efficiency of adenovirus, somatic and male-specific coliphage as representatives of the viral community. For these experiments, MBR treated effluent was spiked with fresh primary effluent to provide the source of wastewater microbes. With ultrafiltration, the somatic and male-specific coliphage recovery efficiency was observed to be 66 (± 20% SD) and 94 (± 17% SD), respectively, as described [17] while the recovery efficiency of adenovirus genomes was 115 (± 128% SD). However, when the ultrafiltrate was filtered through a 0.22 μm membrane filter, the observed recovery was 98 (± 49% SD).

Membrane filtration of all samples was utilized to isolate the virus community (<0.22 μm) from prokaryotic and eukaryotic cells. After the addition of dispersants, raw wastewater influent samples were subjected to a series of successive filtration steps, utilizing 0.8, 0.65, 0.45 and 0.22 μm membrane filters. Through this process alone, the recovery efficiency of somatic and male-specific coliphage was 112 (± 30% SD) and 85 (± 21% SD), respectively.

### DNase efficacy in wastewater samples

**Fig 2:**
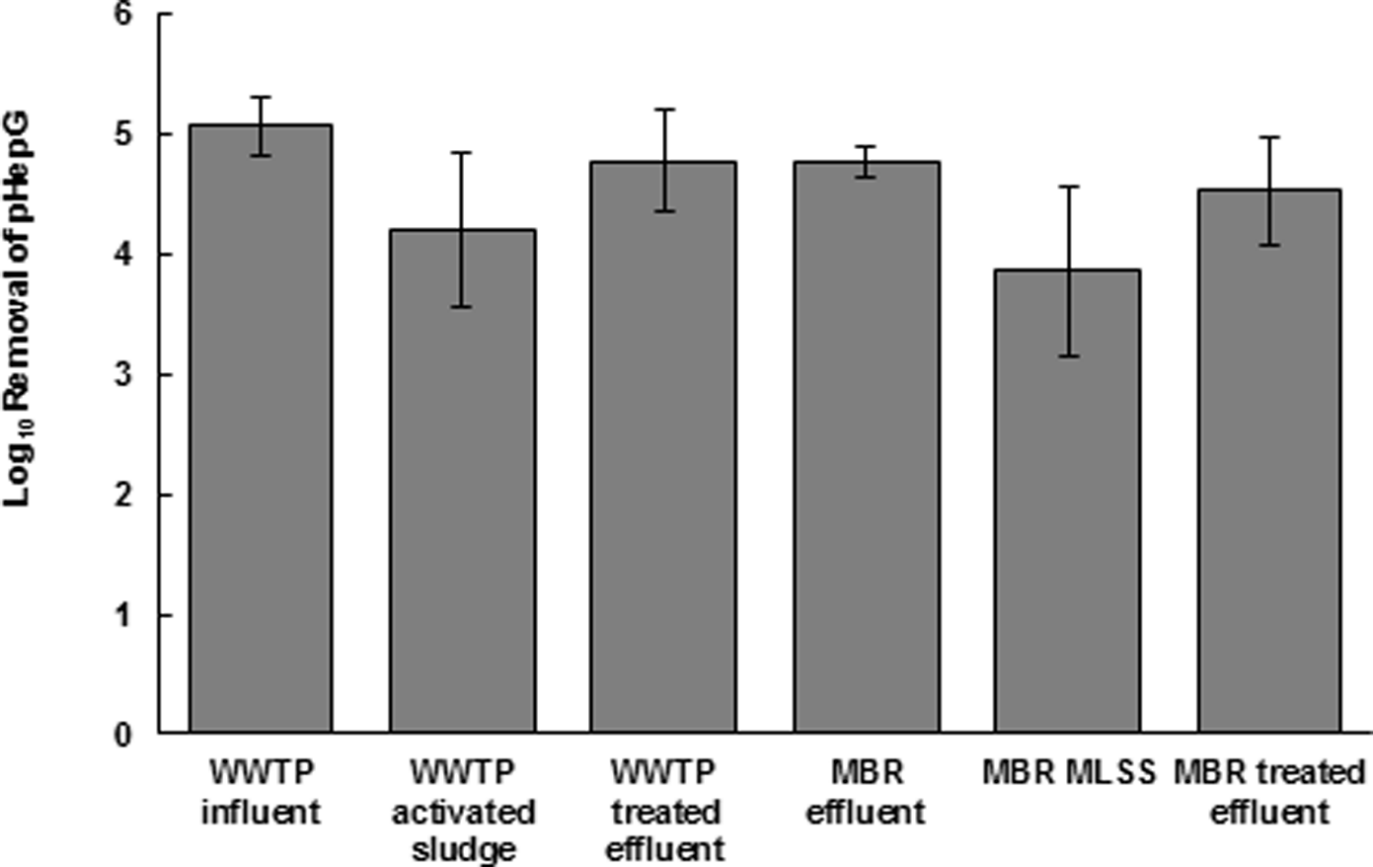
Effects of DNAse digestion on extravirion DNA in various wastewater samples. Values are mean of three replicates. Error bars represent standard deviation. MBR, Membrane bioreactor; MLSS, mixed liquor suspended solids, WWTP, wastewater treatment plant.

The presence of extravirion DNA may limit the sequence coverage and assembly of virion genomes. Therefore, DNase treatment was used to remove this DNA from wastewater samples. Initially, the effects of DNase treatment were assessed using intact AdV virus particles. 4 x 10^4^ and 4 x 10^6^ virus particles were subjected to DNase treatment, along with a set of untreated control reactions containing the same amounts of virus particles. Results revealed that, on average, 54% of AdV DNA detected in the control was resistant to DNase treatment and was not degraded. To determine if DNase was enzymatically active in wastewater samples, which are complex mixtures that could inhibit DNase activity, wastewater samples were spiked with 1 x10^5^ copies of the HepG MiniGene and evaluated for degradation by DNase. Results revealed a 4.54-log_10_ (ranging from 3.86 to 5.07) reduction of the HepG MiniGene signal in wastewater as assessed by qPCR (Fig 2). Analysis of other types of wastewater product (influent, activated sludge/MLSS and UF concentrated effluent samples from a WWTP and small-scale MBR system) also resulted in similar digestion of the HepG MiniGene as determined by qPCR.

### DNA extraction efficiency

The recovery of AdV from the QIAamp DNA Blood extraction kit was 11.9 ± 1%. The impact of genomic DNA loss due to inefficient elution from the silica column was assessed for AdV and PyV. The recovery of virus DNA from the columns was respectively 89.1% (σ = 3.67%, CV = 4.12%) and 135.5% (σ = 41.6%, CV = 30.7%) and there was no significant difference between these recoveries (P = 0.19). Additionally, the addition of the kit protease during the lysis phase of the extraction increased genomic DNA yield for PyV (P = 2.5 x 10^−8^, Mann-Whitney Rank Sum Test), but not AdV (P = 1, Mann-Whitney Rank Sum Test).

### Influence of MDA on circular and linear virus genomes

**Fig 3:**
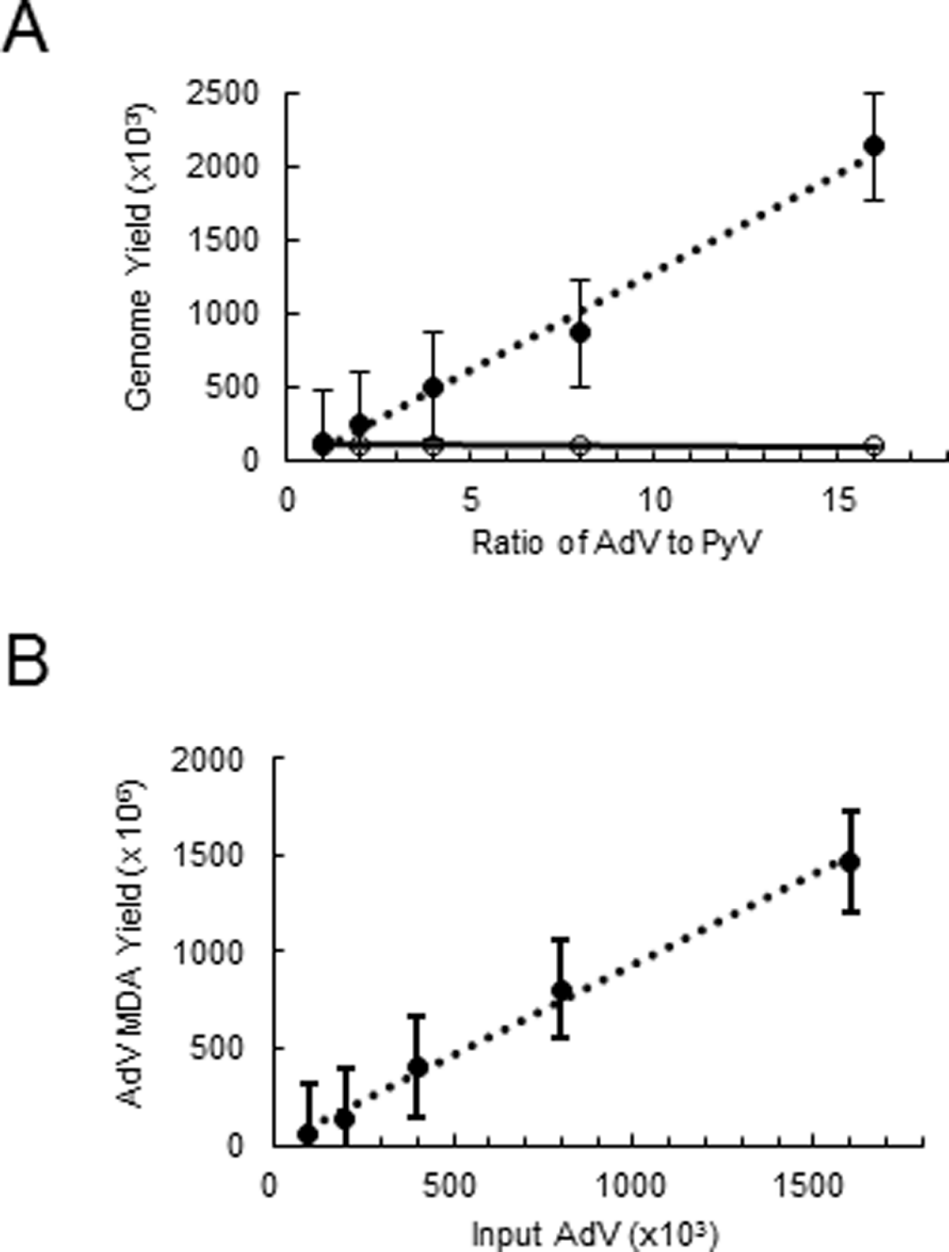
**Detection of increasing AdV with a constant amount of PyV,** A) before MDA and B) after MDA Symbols: AdV (solid circle), PyV (open circle). Error bars represent standard deviation.

**Fig 4:**
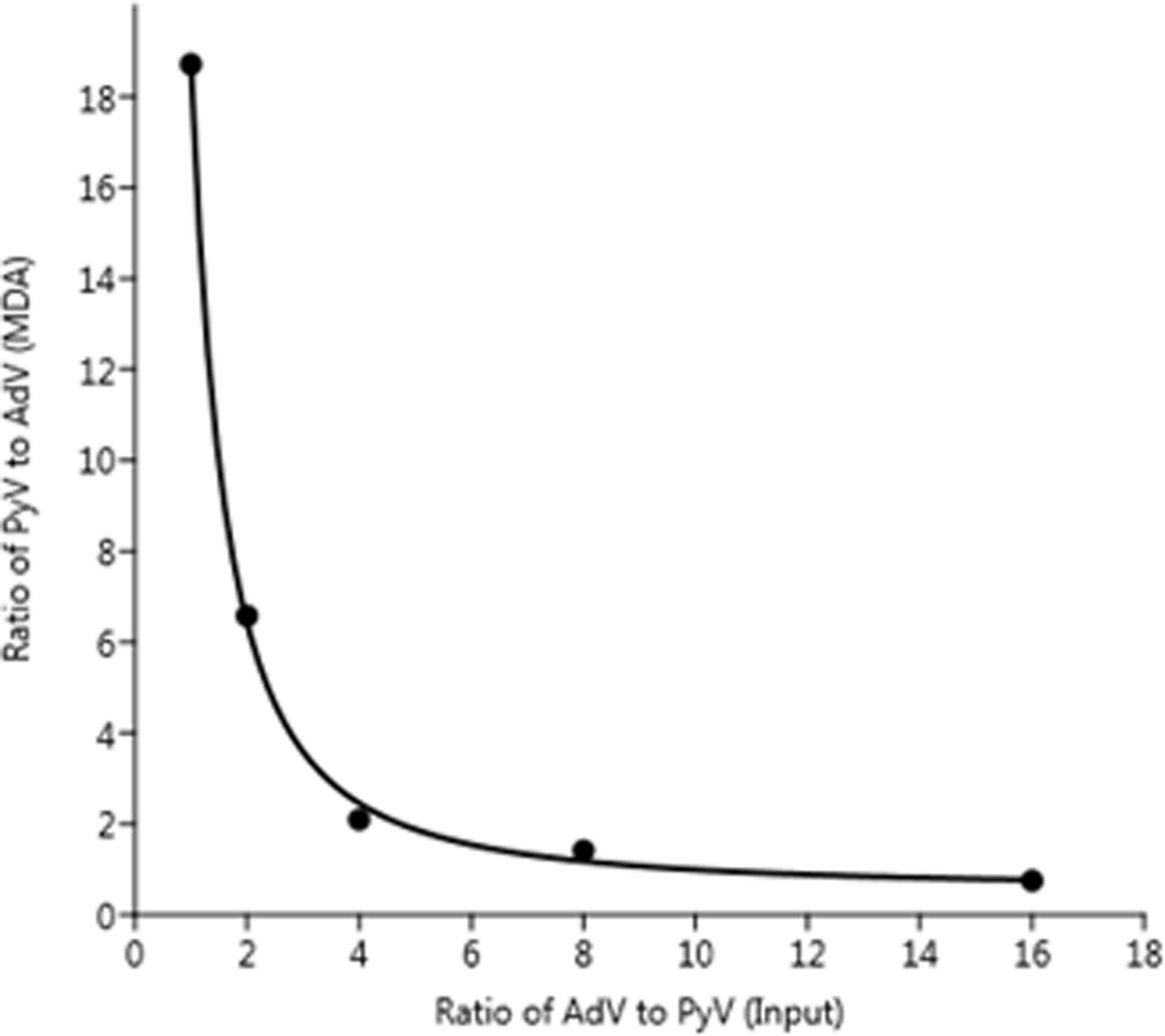
Comparison of normalized input DNA and MDA product ratios. Nonlinear regression of an exponential decay curve was performed using Eq 2, where a = 55.9, b = -1.16 and c = 1.21. The Akaike information criteria (AIC) was 30.384.

Linear regression analysis of AdV quantities prior to MDA indicated a significant, positive relationship between the input concentrations of AdV and the amount measured by ddPCR (y = 1.326x -50,104, R^2^ = 0.9903) (Fig 3A). As expected, the measured quantity of PyV before MDA was close to the targeted addition level, ranging from 97,125 to 107,000 genome copies per reaction. Regression analysis of MDA products showed a significant, positive relationship between the input quantity of AdV and MDA products (R^2^ = 0.9937) (Fig 3B) and the slope indicated a production rate of 941-fold. By contrast, the quantity of PyV MDA product was nearly 5-logs greater than the quantity of input PyV (i.e., 1 x10^5^ genomes). Nevertheless, the quantity of PyV MDA products decreased exponentially as a function of increasing quantities of AdV. To examine this relationship, nonlinear regression analysis (Fig 4) was performed using Eq 2 and the quantity of AdV needed to overcome MDA bias was estimated using Eq 4. Approximately 11.9-fold more AdV than PyV in the input DNA was needed to completely mitigate MDA bias.

The performance of MDA of equal quantities of AdV and PyV was also assessed in wastewater. Linear regression showed MDA of AdV was positively correlated with the concentration of input AdV (y = 1089.2x -5661.1, R^2^ = 1.0) and the slope was similar to Tris buffer described above (cf. 941 vs. 1089) and suggested robust detection of AdV by MDA despite the preference of φ29 DNA polymerase for viruses with small, circular DNA genomes.

### Total recovery of AdV

These data show that the recovery of AdV was affected by DNase treatment, DNA extraction and MDA. To demonstrate the impact of these processes, Eq 5 was used to approximate the total recovery of AdV in a sample containing competing small, circular DNA viruses:

$$\left\{R_{\left(Conc.\right)}=0.98\right\}*\left\{R_{\left(DNase\right)}=0.54\right\}*\left\{R_{\left(QIAamp\right)}=0.13\right\}*\left\{R_{\left(MDA\right)}=\frac{1}{11.9}\right\}=0.0058$$

This calculation suggests the maximum total recovery for a small circular DNA virus would be 173-fold greater than AdV if its concentration was identical to AdV and its process recoveries were 100%.

### Influence of MDA on deep sequencing of wastewater viruses

The negative exponential model proved in a simple system that MDA preference for small, circular viruses could be mitigated by altering the ratio of viruses prior to amplification. However, does this conclusion apply to a complex system such as wastewater known to contain thousands of different viruses? To answer this, MDA followed by deep sequencing was performed on triplicate wastewater DNA viruses serially diluted in (10-fold intervals referred to as d0, d1, d2, d3, d4, and d5 for the lowest to highest dilutions) in Tris buffer. The range of DNA concentrations used for MDA was 30 pg, 3 pg, 300 fg, 30fg, 3fg and 0.3 fg. These DNA estimated concentrations are respectively equivalent to 4–7 x 10^5^, 4–7 x 10^4^, 4–7 x 10^3^, 4–7 x 10^2^, 4–7 x 10^1^, 4–7 x 10^0^ wastewater viruses because most of them contain genomes ranging from 40,000 to 70,000 bp [32]. All 6 MDA reactions generated 1 to 2 micrograms of DNA (S1 Fig).

The average number of paired-end reads and corresponding standard deviation for the dilutions were 1.87 x 10^6^ ± 4.6 x 10^5^ and ranged from 1.5 x 10^6^ ± 3.4 x 10^5^ (d5) to 2.5 x 10^6^ ± 5.5 x 10^5^ (d2) (Part A in S2 Fig). Approximately 27.5% ± 5.1% of the paired-reads were used by Velvet software for the assembly of virtig with lengths of at least 500 bp (Part B in S2 Fig). The number of virtigs generally decreased with higher dilutions from 1702 ± 201, 1299 ± 260, 1526 ± 158, 615 ± 181, 395 ± 65, and 359 ± 410 (Part C in S2 Fig). There was a positive, linear relationship between the concentration of DNA used for MDA and the number of virtigs (Pearson *r* = 0.93, *p* = 0.007). There was no significant difference in the maximum lengths of the virtigs (F-statistic = 1.339, p = 0.3129) throughout the dilutions: they ranged from 12966 ± 4155 to 21253 ± 3369 nucleotides (Part D in S2 Fig).

The N50 steadily increased from 339 ± 5.7 to 1,575 ± 91 for d0 to d3 and then decreased at the higher dilutions (Part E in S2 Fig) and the GC content dramatically decreased from 45% ± 1.0 to 38% ± 0.0 for d0 and d3 before leveling off (Part F in S2 Fig). Pearson correlation analysis revealed GC content was negatively correlated with N50 (*r* = -0.86, *p* = 0.029) (S1 Table). Sequence coverage increased with decreasing concentrations of DNA, ranging from 35.9-fold ± 84.7 to 176.2-fold ±248.0 (S3 Fig). It was negatively correlated with DNA concentration (*r* = -0.94, *p* = 0.005) and the number of virtigs (*r* = -0.95, *p* = 0.003) (S1 Table). A rarefaction analysis was also conducted to determine MiSeq sequence depth. The results revealed that 1000 to 2000 reads resulted in a plateau of virus diversity, up to 22 orders/families (S7 Fig)

The virtig sequences were compared by NMDS of tetranucleotide frequencies to ascertain global differences between the dilutions (S4 Fig). The first axis separated the lowest dilutions (d0, d1, d2 and d3) from d4 and d5, which were dispersed in the plot, and the second axis distinguished d0 and d1 from d2 and d3 as well as the replicates of d4 and d5. This approach, therefore, grouped the virtigs into two major classes: Class I contained d0 and d1 virtigs as well as Aw (orange bubble in S4 Fig) and Class II contained d2 and d3 virtigs. These results together with the analysis above suggested there were two major classes of viruses present in the wastewater sample. Class 1 contained small, GC-rich virtigs with relatively lower sequence coverage and Class II contained large, AT-rich virtigs with higher levels of coverage.

Functional analysis of the virtigs using SEED Subsystems in MG-RAST showed that only bacteriophage proteins were detected in the d0 wastewater sample (S5 Fig) and the dominant protein type identified were bacteriophage major capsid proteins. In addition, no rRNA genes were detected in any of the dilutions.

### MDA bias influenced virus taxonomy

**Fig 5:**
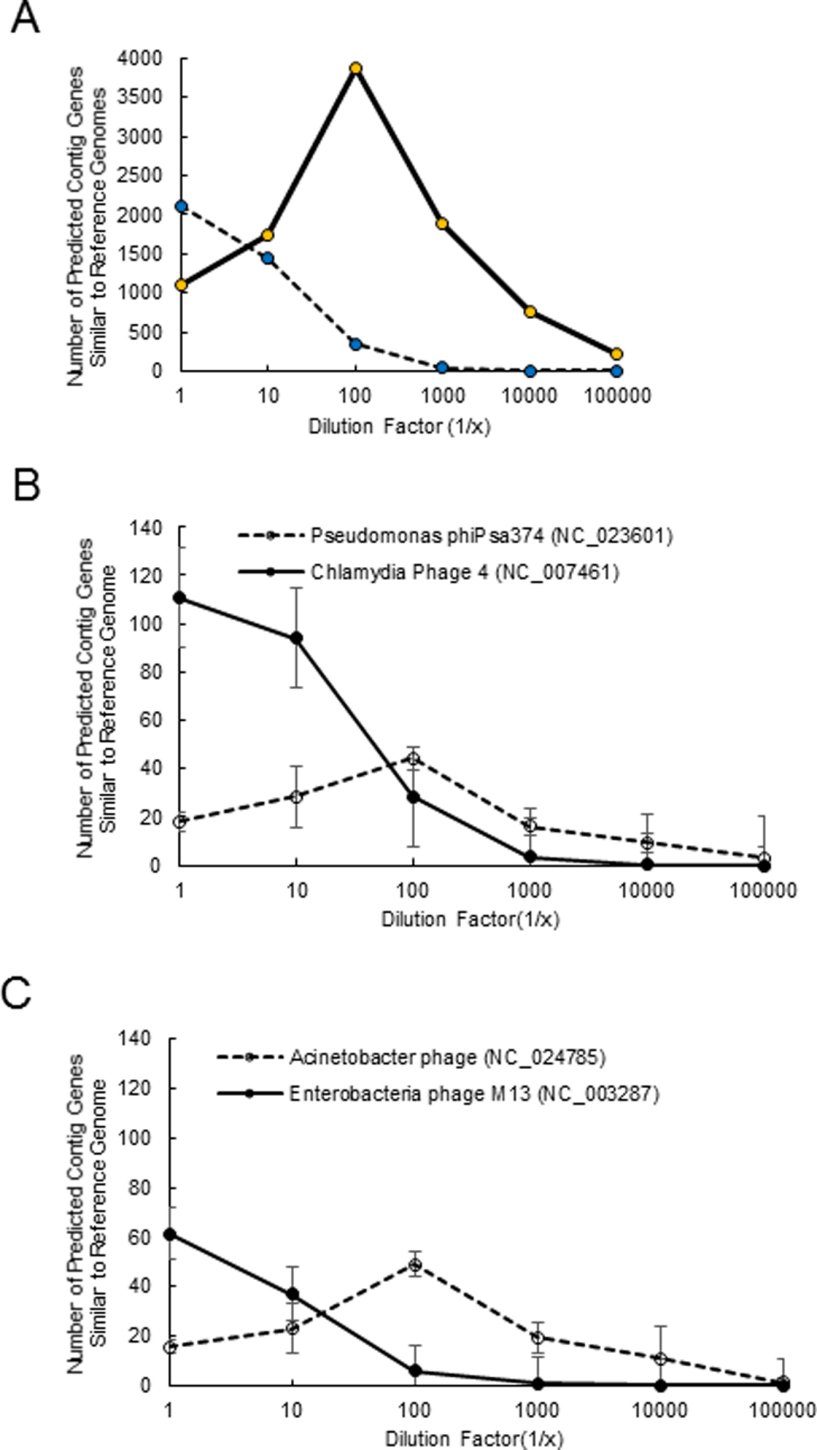
Comparison of relative abundance of bacteriophages with linear and circular genomes. A) ssDNA (blue circle) and dsDNA (orange circle) groups. B) *Chlamydia* phage 4 (NC_007461) (solid circle), vs. *Pseudomonas* phiPas374 (NC_0234601) (open circle). C) *Enterobacteria* phage M13 (NC_003281) (solid circle) vs. *Acinetobacter* phage (NC_024785) (open circle). Error bars represent standard deviation.

The distribution of virus groups in the wastewater sample appeared to be driven by MDA bias as described for the mixing experiments of PyV and AdV. The undiluted (d0) wastewater contained primarily ssDNA viruses that decreased with successive DNA dilutions (Fig 5A), while dsDNA viruses increased in abundance. This was illustrated by comparing select circular and linear viruses identified in d0-d5 (Fig 5B and 5C). Here, the small circular *Chlamydia* and *Enterobacteria* M13 bacteriophages were more abundant at the lower dilutions when compared to larger, linear *Pseudomonas* and *Acinetobacter* bacteriophages.

**Fig 6:**
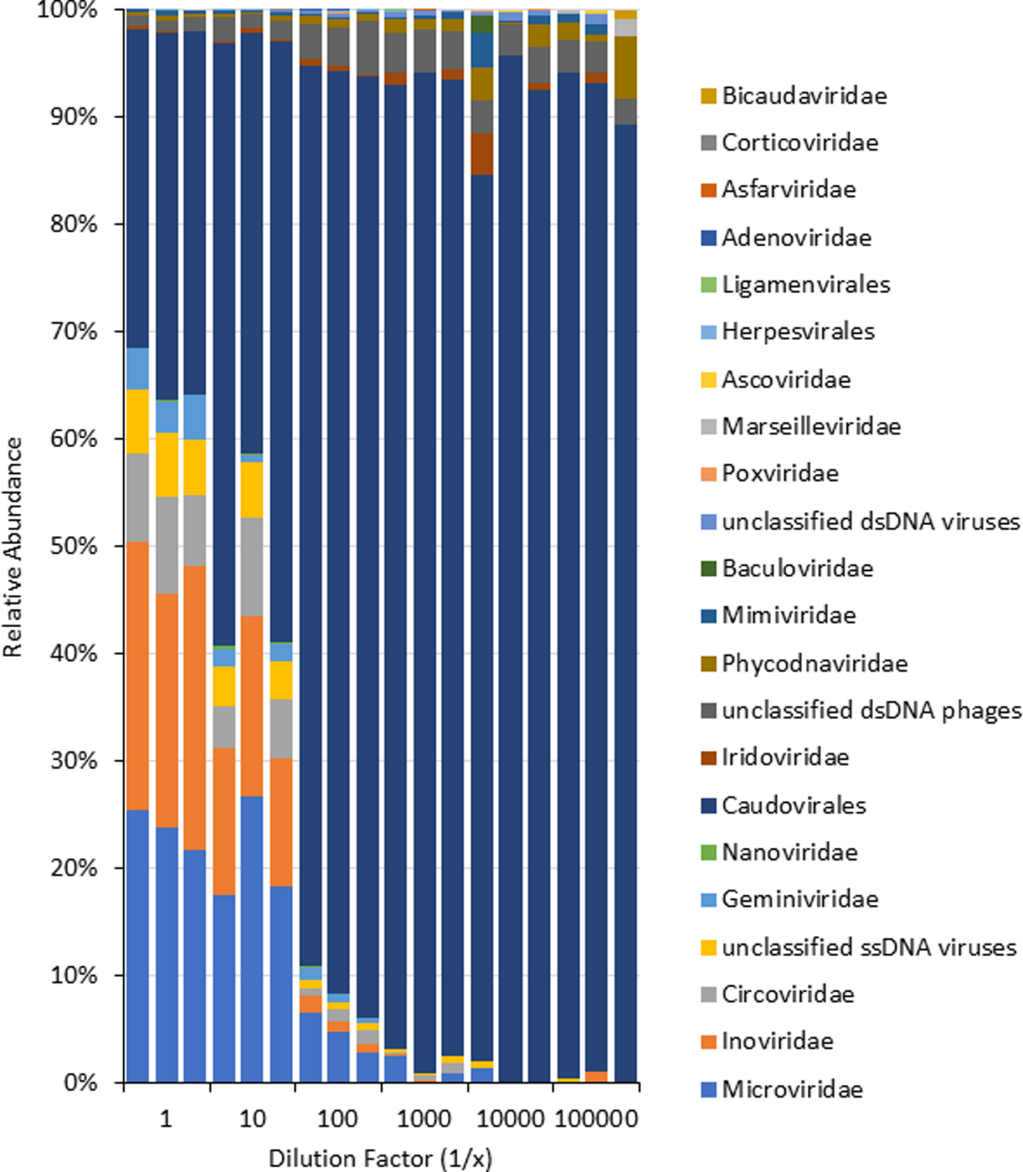
Relative abundance of virus groups and families in a dilution series of a single wastewater sample.

The average number of taxonomically-classified virtigs for d0, d1, d2, d3, d4, and d5 were 47.3 ± 0.72, 53.3 ± 0.67, 59.9 ± 1.73, 61.3 ± 0.17, 36.3 ± 14.6 and 22.7 ± 12.2. Taxonomic analysis revealed 22 virus orders/families and unclassified virus groups (Fig 6) with a host range spanning Eukaryotes, Prokaryotes and Archaea (S2 Table). Family level characteristics (genome size and structure, viral particle size and shape, mode of transmission) of the virtigs observed in d0-d5 are listed in S2 Table. Analysis at the family level (Fig 6) showed that the *Microviridae*, *Inoviridae*, *Circoviridae*, unclassified ssDNA viruses, *Geminiviridae* and *Nanoviridae* decreased in relative abundance with higher dilutions of DNA. The *Microviridae* (genome size = 4.4 to 6.1 Kb), *Inoviridae* (4.5 to 8Kb), *Circoviridae* (1.8 to 3.8Kb) and *Nanoviridae* (1Kb) contain circular ssDNA genomes. By contrast, *Caudovirales*, which have large, mostly linear dsDNA genomes and are comprised of *Myoviridae*, *Siphoviridae* and *Podoviridae*, increased and dominated the relative abundance profile.

*Caudovirales* was the dominant virus group in wastewater dilutions d2-d5. As detailed in S3 Table, the percentage of overall virtigs assigned to *Caudovirales* increased with dilution before plateauing. The percentages of all viruses identified as *Caudovirales* in d0 and d1 are significantly less than d2-d5 (ANOVA, P < 0.001). Within the *Caudovirales*, the distribution of virtigs to the families *Myoviridae* (ANOVA on Ranks, P = 0.06), *Siphoviridae* (ANOVA on Ranks, P = 0.704) and *Podoviridae* (ANOVA, P = 0.256) remained consistent throughout the dilution series.

A comparison of d0 and Aw revealed striking similarities in the distribution of viruses in wastewater. For example, the percentage of ssDNA viruses in d0 and Aw was 63.1 and 63.2 and the percentage of dsDNA viruses was 33.0 and 30.3, respectively. The percentages of *Caudovirales* for d0 and Aw were 33.3 and 26 and the percentages for *Myoviridae*, *Siphoviridae*, *Podoviridae*, and unclassified *Caudovirales* were 48.3 and 38, 21.7 and 31, 27.7 and 31, and 3.1 and 1 (S3 Table).

### Selected viruses of interest

Several of the families identified in the d0-d5 dilutions of the wastewater sample (Fig 6) contained virtigs that were similar to human pathogens (S2 Table). Virtigs showed similarity to proteins of the Human cyclovirus of the *Circoviridae*, and human herpesvirus 4 of the *Herpesviridae*. *Adenoviridae* and *Poxviridae* contain human pathogens, but d0-d5 virtigs were similar to proteins of animal pathogens in these groups.

Virtigs were similar to “giruses”, that is, viruses with very large genomes (>0.5Mb), including *Acanthamoeba polyphaga mimivirus* (1.18Mb), *Acanthamoeba polyphaga moumouvirus* (1.02 Mb), *Cafeteria roenbergensis* virus BV-PV1 (0.62 Mb), *Megavirus chiliensis* (1.26Mb), *Megavirus lba* (1.23Mb), *Pandoravirus dulcis* (1.92 Mb), *Pandoravirus salinus* (2.4 Mb) and *Pithovirus sibericum* (0.61 Mb). Bioinformatic analysis also revealed virophages that infect giruses, including Zamilon virophage (17.3 Kb, dsDNA circular) and three Sputnik virophage variants (18.3Kb, dsDNA circular). Many of the these giruses have been implicated in pneumonia and other respiratory illness [33].

Interestingly, a newly proposed human-specific fecal indicator [34] referred to as crAssphage was similar to several wastewater virtigs (S6 Fig). A crAssphage coverage map of d2, d3, d4 and Aw indicated 96.9% of the genome was observed in wastewater (S6 Fig).

### Principal component analysis of correlated features

**Fig 7:**
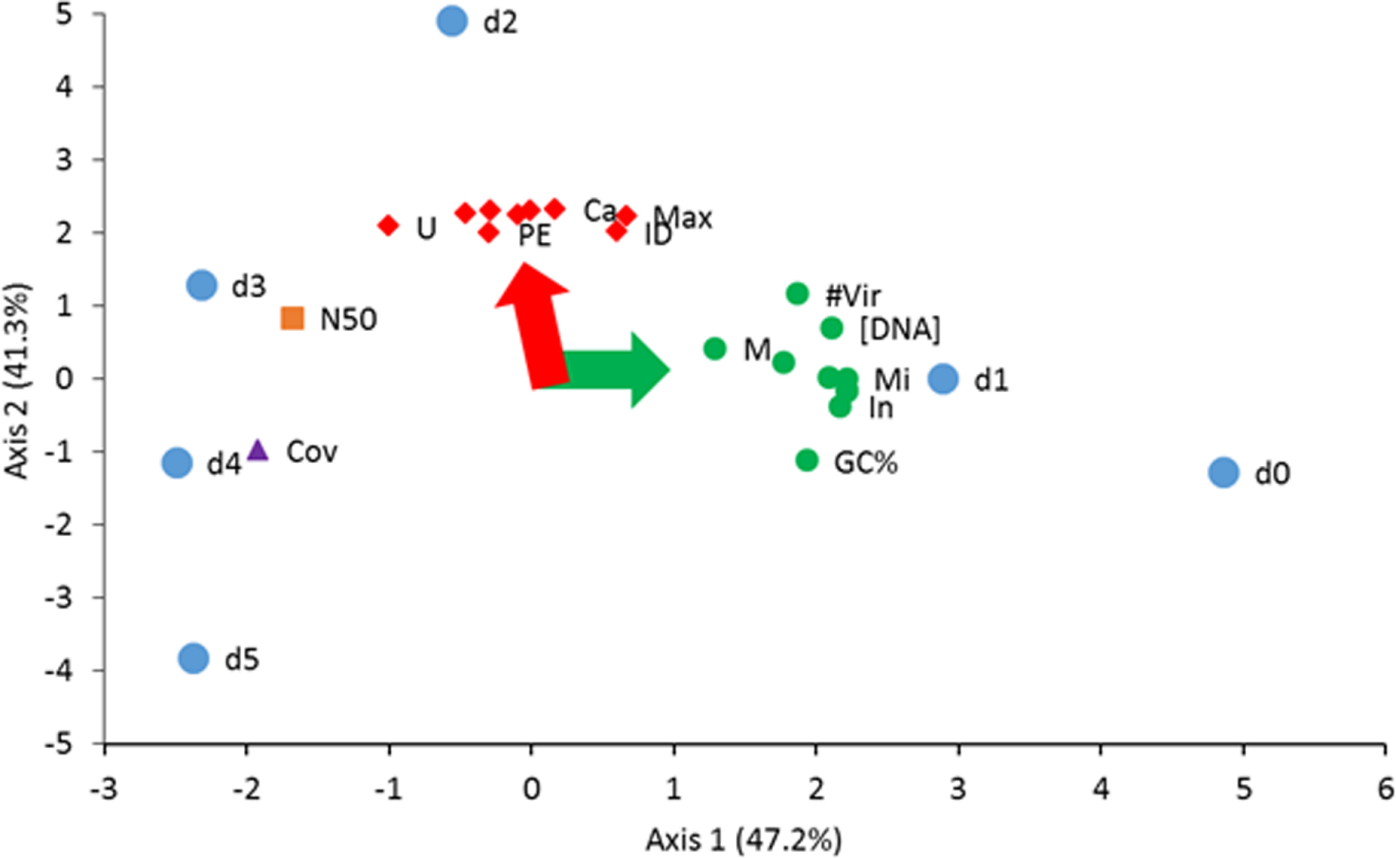
PCA and biplot analysis of molecular parameters and taxonomic units. The ~90° angle between the red and green arrows represent the general trend of the column loadings (red diamonds, green circles, brown square and purple triangle). Specifically, the relationships between the column loadings is represented by the angle between them centered at 0,0 and extending to the position of the data point in the cartesian plot. Abbreviations: sequence coverage (Cov), median virtig length (N50), unclassified dsDNA viruses (U), paired-end reads (PE), *Caudovirales* (Ca), BLAST identified virtigs (ID), maximum virtig length (MAX), *Mimiviridae* (M), number of virtigs (#Vir), log10 DNA concentration used for MDA ([DNA]), *Microviridae* (Mi), *Inoviridae* (In), the percent of the sequence containing G and C nucleotides (GC%), and d0 to d5 (dilutions). Colored symbols: PE reads sequenced, PE reads assembled, maximum virtig length, BLAST identified virtigs, *Caudovirales*, unclassified ds DNA phages, *Phycodnaviridae*, *Iridoviridae*, and unclassified dsDNA viruses (red diamond); DNA concentration for MDA, number of virtigs, GC%, *Inoviridae*, *Microviridae*, *Circoviridae*, unclassified ssDNA viruses, *Geminiviridae*, *Mimiviridae*, *Nanoviridae* (green circle); sequence coverage (purple triangle); N50 (brown box); and dilutions (blue circle).

To better understand the relationships between sequence analysis parameters shown in S2 Fig and virus taxonomy shown in Fig 6, PCA was performed on a correlation matrix comparing the DNA concentration used for MDA, the number of paired-end reads sequenced, the number of paired-end reads assembled, the maximum virtig length, the number of virtigs, the number of BLAST identified virtigs, N50, %GC, sequence coverage per base as well as the top 12 most abundant virus groups and families identified in the dilutions (Fig 7). There was a significant relationship between the Axis 1 separation of d0, d1 from d3, d4 and d5 and the loadings of Class I small circular viruses, including *Microviridae*, *Inoviridae*, *Circoviridae*, *Mimiviridae* Sputnick virus, *Nanoviridae* and unclassified ssDNA viruses. In addition, this separation was associated with the sequence analysis parameters including the number of virtigs (“#Vir”, Fig 7), the concentration of DNA used for MDA (“[DNA]”) and the GC content. Axis 2 was associated with the separation of d2 from the other dilutions and was associated with loadings of the number of paired-end reads sequenced (“PE”), the number of paired-end reads assembled, the maximum virtig length (“Max”), the number of BLAST identified virtigs (“ID”), *Caudovirales* (“Ca”), *Iridoviridae*, *Phycodnaviridae*, and unclassified dsDNA viruses and bacteriophages. The separation of d3 and d4 were respectively associated with N50 and sequence coverage per base and d5 did not have any positive relationships with any of the parameters.

## Discussion

High-throughput sequence analysis is a powerful tool for uncovering the assemblage of viruses in environmental samples. While previous researchers have evaluated the influence of many methodological factors on viral metagenomic results [10, 35–38], we have quantified the potential bias of the different steps in the overall process using controlled manipulations coupled to modeling, and have demonstrated an effective approach for mitigating the bias associated with MDA of wastewater samples using dilution. One challenge for preparing samples for viral metagenomic analysis is initial concentration of samples that may have a dilute viral community. Hollow fiber ultrafilters have been used widely in the field of environmental virology. Using this strategy, we were able to recover 66% (± 20% SD) and 94% (± 17% SD) of culturable somatic and male-specific coliphage, respectively. In addition, we observed a 115% (± 128% SD) recovery of adenovirus genomes using an MPN-PCR approach, and could further reduce the variability of those measurements by filtering the ultrafiltrate through 0.2 μm membrane filter (98% ± 49% SD). Using the 3 viruses examined here as representatives of the viral community, it is probable that the majority of viruses <0.22 μm will be carried through to the next step of sample processing.

Another challenge in preparing samples for viral metagenomic analysis is removal of contaminating prokaryotic and eukaryotic cells. This can be achieved by simply using size restrictive membrane filters to exclude cells. However, viruses can adhere to organic matter [39] and form aggregates [9] in appropriate conditions, which can result in their inadvertent removal during simple filtration procedures. The use of surfactants has been shown to reduce adhesion and aggregation properties of poliovirus in water [40], hence the dispersants used in the elution solution with the hollow fiber ultrafilters was added to wastewater samples prior to membrane filtration. Using the somatic and male-specific coliphage as representatives of the virus community, we were able to recover the majority of these coliphage (111% and 85%, respectively) in raw wastewater influent by adding sodium polyphosphate and Tween 80 before membrane filtration. This procedure was effective in enriching for the viral component of the microbial community as the virtigs produced from sequencing were similar to phage proteins and did not match any bacterial derived genes after analysis in MG-RAST (S5 Fig).

Another important part of the virus collection, concentration, and extraction procedure is selectively removing DNA from extraviral contaminants. DNase treatment is especially efficient in removing these extraviral DNA from any virus preparations. However, this approach also comes at a cost, as shown in our controlled experiments, 46% of the AdV spiked into the sample were susceptible to DNase digestion and thus lost, contributing the overall DNA performance recovery of the method. Nevertheless, enzymatic activity remained intact in wastewater samples. The resulting DNase digestion of the sample prior to any MDA and/or sequencing reaction resulted in the loss of all bacterial DNA. Furthermore, sequence analyses of MDA samples also showed no bacterial DNA sequences detected (S5 Fig), and that only viral genes were detected using SEED analyses. The efficiency of the DNase treatment described in this current study showed that this procedure was able to digest extraviral DNA from wastewater influent, concentrated effluent from municipal WWTP, and small-scale MBR systems samples (Fig 2). Despite some loss in input DNA, DNase treatment is critical in extracting highly purified virus samples fit for downstream MDA reactions and viral metagenomic analyses, especially in complex samples containing high levels of extraviral DNA.

Extraction of genomic material is typically performed using a commercial kit due to the ease of use and low cost. Ideally, genomic extraction methods should isolate nucleic acid with high integrity, purity and in sufficient quality evenly across all types of genomes (i.e. RNA vs DNA, linear vs. circular and single vs. double stranded). A number of strategies are utilized, including physical or chemical disruption, silica columns, magnetic beads and alcohol precipitation. In turn, the type of strategy used often results in a compromise between yield and purity [41]. To understand the effect of genome recovery on various genome types representative of a wastewater viral community, adenovirus and polyomavirus was investigated. Using the QIAamp DNA Blood Mini Kit as described in EPA’s Method 1615 and well quantified virus preparations, 14% (± 0.5) of adenovirus genomes can be recovered. Adding the protease step to the extraction process increases the total mean recovery to and 15 (± 3)% for adenovirus, but these increases are not statistically significantl using Mann Whitney Rank Sum Test. These results suggest that some viruses are not extracted efficiently, thus allowing some potential bias to be propagated through the remaining processing steps and potentially skewing the observed distribution of virus groups in the viral community.

Whole genome amplification, has been useful in studying genomes in low abundance, but it is known to introduce stochastic and systematic biases and therefore not recommended for quantitative inferences [12] until the source(s) of biases have been identified and additional efforts made to minimize those biases. In viral metagenomics analyses, an unusually high representation of small (< 10 Kb) circular DNA viruses (e.g., *Microviridae* family of viruses) was identified in oceanic samples [42] and wastewater samples [6]. This is in contrast to studies that use sequence-independent methods (i.e. pulse field gel electrophoresis) to describe viral diversity in oceanic samples [43, 44] and activated sludge of wastewater [32]; these reports suggest that the most abundant viruses in these matrices contained genomes that were 40–70 Kb. In our study, we further showed that the use of MDA for viral metagenomics indeed revealed a preference amplifying small single stranded DNA viruses (e.g. *Microviridae*) (Figs 5A and 6). However, through our competition experiment presented in Fig 4, we demonstrated MDA bias was a function of the ratio of linear to circular virus genomes and bias could be mitigated by increasing the input DNA concentration of linear genome viruses. This model predicted serial dilution of wastewater would change the ratio of linear to circular virus genomes and overcome MDA bias. Wastewater samples that contain undiluted or were diluted 10-fold had an overabundance of small ssDNA sequences as shown in Figs 6 and 7. However, as the DNA input was diluted down at least 100-fold, the dominance of sequences from small circular ssDNA diminished and viruses with large dsDNA genomes emerged as dominant members of the community reflective of sequence-independent data [32]. Results observed from higher dilutions behaved in a more stochastic fashion presumably due to the distribution of viral genomes described by an underlying probability function (e.g., Poisson). Diluting the starting DNA down to approximately 100-fold resulted in no changes in the amount and lengths of virtigs detected (S2 Fig). However, higher dilutions resulted in a gradual decrease in the number of virtigs. Interestingly, the GC% content was skewed from highly GC rich (~45%) at the undiluted sample, to lower GC content (~38%) in the 1/100 diluted sample and remained unchanged for the higher dilutions. Despite the biases caused by MDA it is a powerful tool for revealing less abundant ssDNA circle viruses unique to environmental matrices [42]. Our analysis showed that the undiluted wastewater sample revealed *Microviridae*, *Inoviridae*, *Circoviridae*, *Geminiviridae* and *Nanoviridae*, which were not frequently observed in the higher dilutions.

Bioinformatic analyses of environmental viral community sequences obtained from massively parallel sequencing approaches can be daunting. Reports to date have shown that in marine viral metagenomes, up to 91% of viral sequences are unknown or unclassified [3], while viral sequences identified from other environmental samples have also been difficult to taxonomically classify [16]. Analysis of wastewater virtigs indicated more than half of them were not similar to any known virus reference genome. The high percentage of unknown sequences generated from culture-independent virus samples of environmental matrices suggest that viral abundance and diversity may be the largest with the most complex community structure found in the environment. Despite these uncertainties, new statistical approaches have been developed to include unknown viruses. For example, a new bacteriophage, crAssphage, was discovered using a reference-independent cross-assembler [45]. In the study herein, the crAssphage sequence was shown to be similar to virtigs in wastewater (S6 Fig). Di-, tri- and tetra nucleotide frequencies have been used to compare biomes containing known and unknown viruses [46]. This approach was successfully used to compare all the viruses in the wastewater dilutions (S4 Fig). Thus, careful and thorough bioinformatic analyses are essential for understanding the abundance and diversity of viruses present in a given biome.

**Fig 8:**
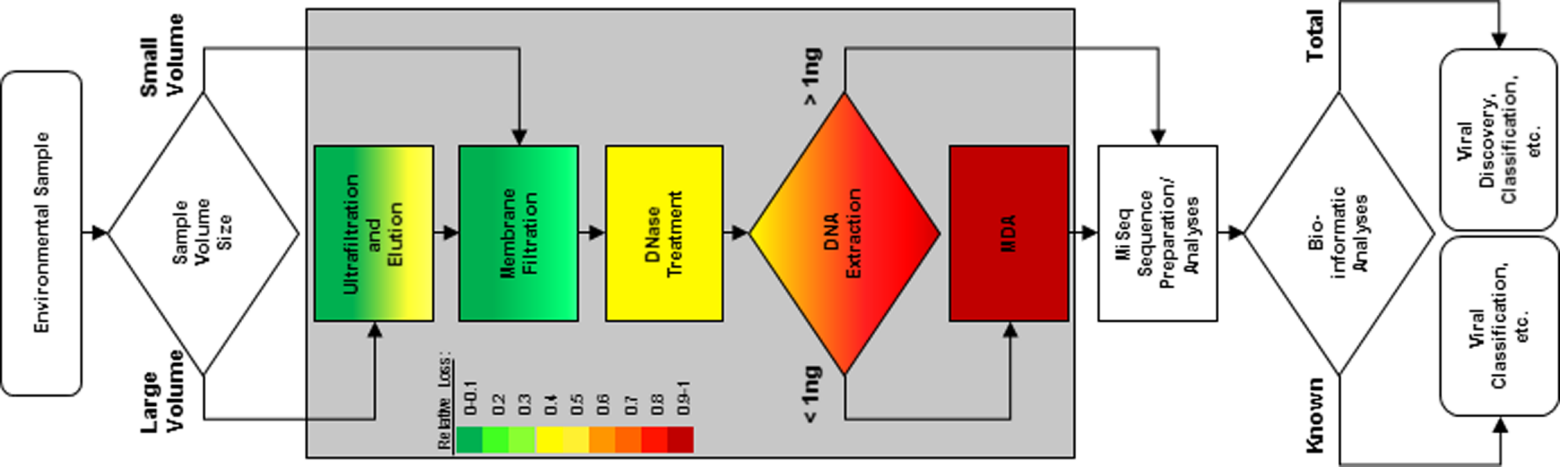
Sample to sequence work flow diagram illustrating the relative losses identified at each of the steps involved for wastewater viral metagenomic analysis. Relative losses were quantified in specific steps inside the shaded area. A heat map was generated to graphically represent the losses measured at the ultrafiltration and elution, membrane filtration, DNase treatment, DNA extraction and MDA steps. Heat map legend is shown on the left side of the shaded box.

These results are relevant to on-going water reuse research. Rapid monitoring of treatment efficacy with respect to viral pathogens is critical to minimizing risks associated with water reuse, but direct assessment of viral pathogen densities in treated water is impractical given their low and variable levels [47]. Alternative viruses have been used as surrogates for assessing treatment, but typically rely on spiking of targets to intermittently validate performance or measuring bacteriophage associated with human fecal bacteria which also may be found at relatively low levels. An alternative surrogate approach would target the bacteriophage generated during biological treatment of wastewater which may afford the greatest potential for evaluating log removal in the finished water. The overall metagenomic analysis (Fig 6) clearly indicates that bacteriophage dominate the wastewater viral community, and the dilution results suggest that large dsDNA bacteriophage are the most common members of these wastewater viral communities. On-going metagenomic analysis of a range of wastewaters through the water reuse treatment chain will define the consistency of these results, but this study predicts that dsDNA bacteriophage rather than small ssDNA bacteriophage are the best targets for surrogate development. In particular, members of the *Caudovirales* are likely candidates given that they are the most abundant group identified in wastewater and their consistent distribution across families is retained even when MDA preference for small circular viruses is evident. Overall, the method presented herein provides a well characterized approach for obtaining effective recovery of bacteria-free viral DNA from wastewater. Fig 8 shows a flow diagram that provides how future studies focused on conducting viral metagenomics analyses of environmental samples (e.g., wastewater) should be performed to achieve high quality DNA samples that have been processed to minimize contaminating bacterial DNA as well as minimizes biases introduced during the MDA reaction step. The diagram also identifies the relative losses characterized each of the procedures developed and applied to conduct a viral metagenomic analysis of wastewater samples, which assist in estimating how much starting material should be collected in order to extract sufficient amount of samples for viral metagenomic analyses.

Lastly, careful evaluation of bias, especially when MDA is needed to evaluate low biomass samples, can be accomplished through analysis of sample dilutions, resulting in a more accurate description of the dominant members of the viral community. While care should be taken to avoid over dilution and concomitant increases in stochasticity due to low template numbers and potential contamination, the dilution approach can be used by researchers to test the accuracy of metagenomics studies reporting the dominance of small circular bacteriophage form other types of environments.

## Supporting information

S1 Table Pearson correlation of sequence results. Lower quadrant is the Pearson statistic and upper quadrant is the p-value. Yellow areas are significant or borderline significant.
(DOCX)

S2 Table Family level details of viruses identified in d0-d5 of the wastewater influent sample. (DOCX)

S3 Table Taxonomic composition of virtigs similar to *Caudovirales* in d0-d5. Taxonomic compositions were computed using BLASTP with NCBI RefSeq complete viral genomes proteins and a bitscore of 50. *1% of the viruses were RNA viruses.
(DOCX)

S1 Fig MDA generates micrograms of DNA from femtograms of wastewater virus DNA. (DOCX)

S2 Fig Sequencing and assembly results from dilutions of the wastewater sample. A) Number of paired-end read; B) Used paired-end reads; C) Number of virtigs assembled; D) Maximum length of virtigs; E) Velvet N50; F) GC content of virtigs. The mean and standard deviation of triplicate samples are shown.
(DOCX)

S3 Fig Coverage plots for the DNA dilutions. Panels A(d0), B(d1), C(d2), D(d3), E(d4), and F(d5). The mean coverage and standard deviation are shown at the top of each panel.
(DOCX)

S4 Fig Non-metric multidimensional analysis of viral community nucleotide composition bias. Tetranucleotide frequencies were determined using MetaVir2. The diameter of the bubbles represents the relative amount of log_10_ DNA used for each MDA reaction. The orange bubble is data from Aw et al. [6, 7].
(DOCX)

S5 Fig Bacteriophage genes detected in wastewater. Hierarchical classification using SEED Subsystems (function level) was used to describe the genes in the undiluted d0 virtigs. Criteria: A maximum e-value of 1e-5, a minimum identity of 60%, and a minimum alignment length of 15 measured in amino acids for protein. DESeq was used for normalization. The heatmap was clustered using ward with bray-curtis distance metric via MG-RAST pipeline. The heatmap key for the normalized and scaled values is shown at the bottom.
(DOCX)

S6 Fig Genome coverage of crAssphage. Red and blue proteins have known (protein names are vertical below the x-axis) and unknown functions, respectively. The cumulative number of predicted genes similar to each gene of crAssphage is indicated by vertical bars. Abbreviations: Aw is data from Aw et al. [6, 7]; d2, d3 and d4 are dilutions from the current study with replicates indicated by A, B or C.
(DOCX)

S7 Fig Rarefaction analysis of DNA dilutions. (DOCX)
