# Supplementary material for: Reducing inherent biases introduced during DNA viral metagenome analyses of municipal wastewater
Source: PLoS One. 2018 Apr 3;13(4):e0195350. doi: 10.1371/journal.pone.0195350 (PMC5882159; doi:10.1371/journal.pone.0195350)
Supplement: S2 Table — (DOCX) [file pone.0195350.s002.docx]

**S2 Table**

| **Group** | **Taxonomic level** | **Genome Strandedness** | **Genome Structure** | **Genome Size (kb)** | **Host** | **Morphology** | **Physical size (nm)** | **Mode of Transmission** | **Human Pathogens Identified in d0-d5** |
| --- | --- | --- | --- | --- | --- | --- | --- | --- | --- |
| *Microviridae* | Family | ssDNA(+) | circular | 4.4-6.1 | bacteria | icosahedral | 30 nm diameter |  | Not applicable |
| *Inoviridae* | Family | ssDNA(+) | circular | 4.5-8 | bacteria | Rods of filaments | 7 nm diameter, 700-2000 nm length |  | Not applicable |
| *Circoviridae* | Family | ssDNA | circular | 1.8-3.8 | Birds and mammals | icosahedral | 20 nm diameter | Fecal-oral | Human cyclovirus |
| *Geminiviridae* | Family | ssDNA(+) | mono- or bipartite, circular | 2.5-3 (monopartite) or 4.8-5.6 (bipartite) | plants | Twinned, incomplete icosahedral | 22 nm diameter, 38 nm length | Vector = insects | Not applicable |
| *Nanoviridae* | Family | ssDNA (+) | Multipartite: 6-8 circular segments | About 1kb each segment | plants | icosahedral | 18-19 nm diameter | Vector = Aphids | Not applicable |
| *Caudovirales* | Order: 3 Families – *Myoviridae*, *Podoviridae* and *Siphoviridae* | dsDNA | linear | 33-244 | Bacteria, archaea | Head-tail | 60 nm diameter | Passive diffusion through medium | Not applicable |
| *Iridoviridae* | Family | dsDNA | linear | 140-303 | Amphibia, fish, invertebrates | Icosahedral, can be enveloped | 120-350 nm diameter |  | Not applicable |
| *Phycodnaviridae* | Family | dsDNA | linear | 100-560 | algae | Enveloped, icosahedral | 100-220 nm diameter | Passive diffusion through water | Not applicable |
| *Mimiviridae* | Family | dsDNA | linear | 1200 | amoeba | icosahedral | 400 nm diameter |  | *Acanthamoeba polyphaga mimivirus, Acanthamoeba polyphaga moumouvirus* |
| *Baculoviridae* | Family | dsDNA | circular | 80-180 | arthropods | Enveloped, occluded or budded forms | 21 nm diameter, 260 nm length | Fecal-oral route | Not applicable |
| *Poxviridae* | Family | dsDNA | linear | 130-375 | Humans, vertebrates, arthropods | Enveloped, brick-shaped or ovoid | 140-260 nm diameter, 220-45 nm length | Respiratory droplets, direct contact or fomites | None detected (deer, rabbit, bovine, etc. poxvirus detected) |
| *Marseilleviridae* | Family | dsDNA | circular | 372 | amoeba | icosahedral | 250 nm diameter |  | Melbournevirus |
| *Ascoviridae* | Family | dsDNA | circular | 156-186 | insects | Enveloped, bacilliform, ovoidal or allantoid | 130 nm diameter, 200-400 nm length | horizontally by endoparasitic wasps | Not applicable |
| Herpesvirales | Order: 3 Families – *Alloherpesviridae*, *Herpesviridae*, *Malacoherpesviridae* | dsDNA | linear | 120-248 | Fish, vertebrates, mollusks | Enveloped, spherical to pleimorphic | 150-200 nm diameter | Fish: passive diffusion through water; Vertebrates: Contact with lesions and body fluid | Human herpesvirus 4 |
| *Ligamenvirales* | Order: 2 Families – *Lipothirxviridae* and Rudiviridae | dsDNA | linear | 15.9-56 | Archaea | Rod shaped, can be enveloped | 24-38 nm diameter, 410-1950 nm length | Passive diffusion through medium | Not applicable |
| *Adenoviridae* | Family | dsDNA | linear | 34-36 | vertebrates | icosahedral | 90 nm diameter | Respiratory droplets, fecal-oral route | None detected (bovine, fowl, sea lion adenovirus detected) |
| *Asfarviridae* | Family | dsDNA | linear | 170-190 | Pigs | Enveloped, spherical to pleimorphic | 175-210 nm diameter | Vector = arthropods | Not applicable |
| *Corticoviridae* | Family | dsDNA | circular | 10 | bacteria | icosahedral | 56 nm diameter | Passive diffusion through medium | Not applicable |
| Bicaudaviridae | Family | dsDNA | circular | 62 | archaea | Lemon, 2-tailed |  | Passive diffusion through medium | Not applicable |
