# Supplementary material for: Reducing inherent biases introduced during DNA viral metagenome analyses of municipal wastewater
Source: PLoS One. 2018 Apr 3;13(4):e0195350. doi: 10.1371/journal.pone.0195350 (PMC5882159; doi:10.1371/journal.pone.0195350)
Supplement: S3 Table — Taxonomic compositions were computed using BLASTP with NCBI RefSeq complete viral genomes proteins and a bitscore of 50. *1% of the viruses were RNA viruses. (DOCX) [file pone.0195350.s003.docx]

**S3 Table.**

| **Dilution** | **Replicates** | ***Caudovirales***  **(% of all viruses)** | ***Caudovirales*** | | | |
| --- | --- | --- | --- | --- | --- | --- |
|  |  |  | ***Myoviridae***  **(%)** | ***Podoviridae***  **(%)** | ***Siphoviridae***  **(%)** | **Unclassified (%)** |
| d0 | A | 32 | 49 | 21 | 28 | 4 |
|  | B | 35 | 47 | 21 | 29 | 3 |
|  | C | 33 | 49 | 23 | 25 | 4 |
| d1 | A | 56 | 47 | 23 | 27 | 3 |
|  | B | 41 | 45 | 19 | 32 | 4 |
|  | C | 60 | 47 | 19 | 30 | 3 |
| d2 | A | 84 | 38 | 24 | 35 | 3 |
|  | B | 92 | 42 | 21 | 35 | 2 |
|  | C | 91 | 39 | 23 | 34 | 4 |
| d3 | A | 92 | 39 | 22 | 37 | 2 |
|  | B | 91 | 39 | 22 | 37 | 3 |
|  | C | 93 | 52 | 19 | 25 | 4 |
| d4 | A | 71 | 35 | 19 | 43 | 3 |
|  | B | 88 | 42 | 20 | 36 | 1 |
|  | C | 87 | 39 | 24 | 32 | 5 |
| d5 | A | 90 | 51 | 9 | 38 | 2 |
|  | B | 84 | 52 | 10 | 34 | 5 |
|  | C | 85 | 47 | 24 | 24 | 6 |
| AW | single | 26 | 38 | 31 | 31 | 1 |

Taxonomic compositions were computed using BLASTP with NCBI RefSeq complete viral genomes proteins and a bitscore of 50. *1% of the viruses were RNA viruses
