# Supplementary figures and images for: Reducing inherent biases introduced during DNA viral metagenome analyses of municipal wastewater
Source: PLoS One. 2018 Apr 3;13(4):e0195350. doi: 10.1371/journal.pone.0195350 (PMC5882159; doi:10.1371/journal.pone.0195350)

**S2 Fig**


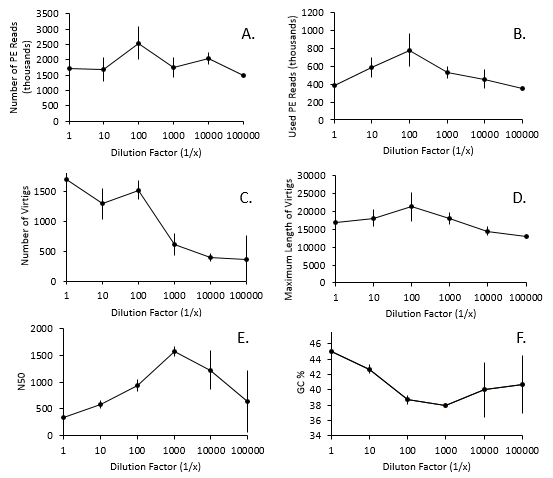

Supplement: S2 Fig — A) Number of paired-end read; B) Used paired-end reads; C) Number of virtigs assembled; D) Maximum length of virtigs; E) Velvet N50; F) GC content of virtigs. The mean and standard deviation of triplicate samples are shown. (DOCX) [file pone.0195350.s005.docx]

**S3 Fig.**


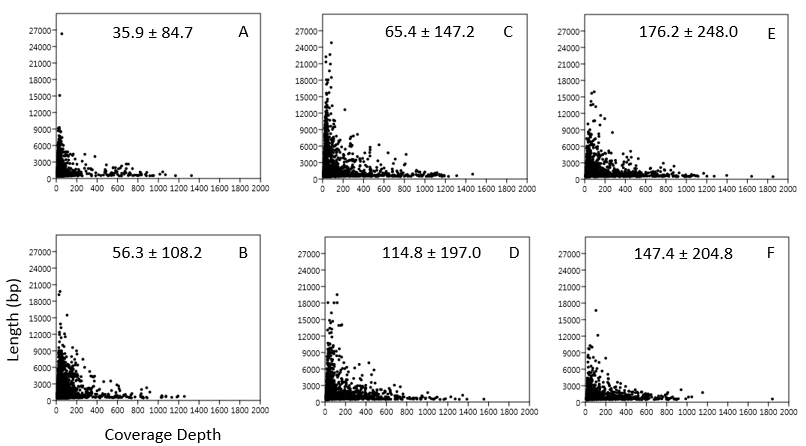

Supplement: S3 Fig — Panels A(d0), B(d1), C(d2), D(d3), E(d4), and F(d5). The mean coverage and standard deviation are shown at the top of each panel. (DOCX) [file pone.0195350.s006.docx]

**S5 Fig.**


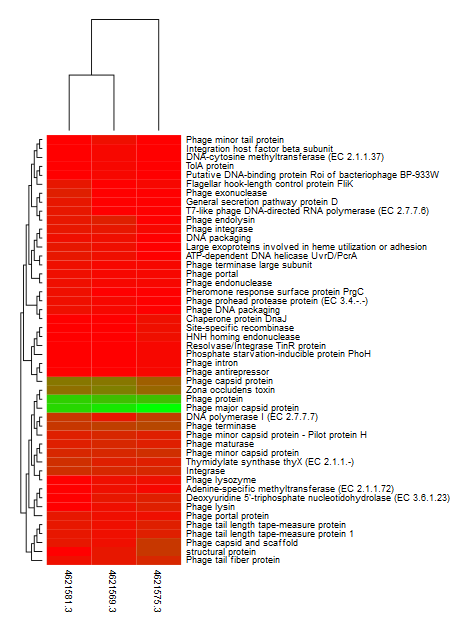


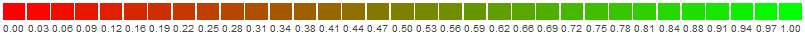

Supplement: S5 Fig — Hierarchical classification using SEED Subsystems (function level) was used to describe the genes in the undiluted d0 virtigs. Criteria: A maximum e-value of 1e-5, a minimum identity of 60%, and a minimum alignment length of 15 measured in amino acids for protein. DESeq was used for normalization. The heatmap was clustered using ward with bray-curtis distance metric via MG-RAST pipeline. The heatmap key for the normalized and scaled values is shown at the bottom. (DOCX) [file pone.0195350.s008.docx]

**S6 Fig.**


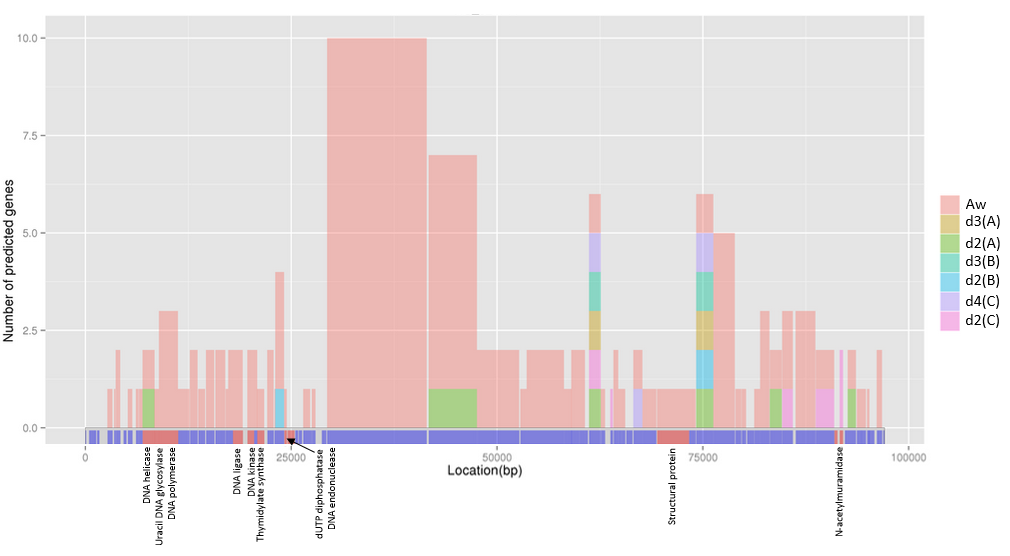

Supplement: S6 Fig — Red and blue proteins have known (protein names are vertical below the x-axis) and unknown functions, respectively. The cumulative number of predicted genes similar to each gene of crAssphage is indicated by vertical bars. Abbreviations: Aw is data from Aw et al. [6, 7]; d2, d3 and d4 are dilutions from the current study with replicates indicated by A, B or C. (DOCX) [file pone.0195350.s009.docx]

S7 Fig.


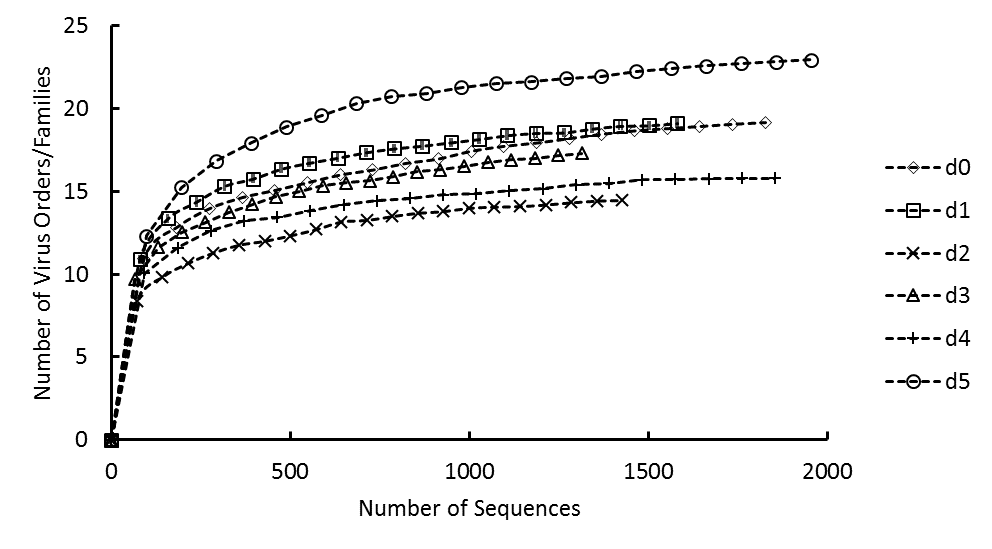

Supplement: S7 Fig — (DOCX) [file pone.0195350.s010.docx]
